# P01.49. Development of quality control methods for some Indian integrative medicines by use of markers

**DOI:** 10.1186/1472-6882-12-S1-P49

**Published:** 2012-06-12

**Authors:** A Mohammad, Swarnlata Saraf, Shailendra Saraf

**Affiliations:** 1 University Institute of Pharmacy, Raipur, India

## Purpose

In the present study quality control methods for two Indian integrative medicines included in the Ayurveda system, Balchaturbhadrika churna and Shyngyadi churna, were established with the help of a standard marker compound. These preparations are widely used in India for the treatment of diarrhea, fever, cough, and asthma.

## Methods

Stated formulations contain the common crude drug named piper longum. As we know that piperine is the key active plant ingredient of the drug and responsible for its therapeutic activity, both formulations have been standardized through high performance thin layer chromatography with respect to this marker compound (piperine). The stationary phase used was precoated silica gel 60F 254. The mobile phase containing toluene and methanol in proportion of 80:10 v/v was used to separate the spot of piperine. The detection of the spot was carried out at 345.5 nm. The proposed HPTLC method can be used for quality control of the raw material as well as the formulations.

## Results

In the chromatogram of the formulations, one spot matched with the Rf value of standard piperine and had the same max (345.5 nm), which indicates the presence of piperine in the formulations. The low percent residual standard deviation values indicate the suitability of this method for routine analysis of piperine in both formulations.

## Conclusion

Rapid, reproducible, and accurate methods for the qualitative and quantitative estimation of piperine in both Ayurvedic medicines have been developed, which can be very useful for the routine quality control analysis of these formulations and to increase their global acceptance.

